# Metastatic Leiomyosarcoma to the Spine Complicated With Thrombocytopenia

**DOI:** 10.4021/wjon481w

**Published:** 2012-08-26

**Authors:** Ali Shirzadi, Doniel Drazin, Neda Shirzadi, Franklin Westhout, Noam Drazin, Xuemo Fan, Frank L. Acosta

**Affiliations:** a Department of Neurosurgery, Cedars Sinai Medical Center, Los Angeles, CA, USA; b Department of Internal Medicine, Division of Hematology/Oncology, Cedars Sinai Medical Center, Los Angeles, CA, USA; c Department of Pathology and Laboratory Medicine, Cedars-Sinai Medical Center, Los Angeles, CA, USA

**Keywords:** Leiomyosarcoma, Spine, Uterine, Thrombocytopenia

## Abstract

Uterine leiomyosarcomas do not frequently metastasize to the bone, and spinal column metastases are even less common. Surgery is the treatment of choice. Adjuvant radiation with or without chemotherapy depending on the extent of disease can be beneficial. We present the case of leiomyosarcoma metastasis to the spine with a previous history of known primary disease complicated by thrombocytopenia. Thrombocytopenia can present surgical challenges, but can also be present concurrently unrelated to the primary disease. A thorough evaluation is needed to rule out any underlying lymphoproliferative disorder or microangiopathic phenomenon.

## Introduction

Intraspinal leiomyosarcoma metastases are rare [1-3]. These lesions often are destructive to the bone and as such can result in burst fractures of the vertebra [3-5]. In addition patients experience compression of the spinal canal either due to direct extension of the mass into the canal or as a result of bony fragments due to destructive process of the lesion on the vertebra. The main treatment for leiomyosarcoma to the spine is surgical decompression and resection. Most patients with paravertebral lesions rarely present simultaneous metastatic lesions in other parts of the body. Adjuvant chemotherapy is recommended for recurrent or metastatic disease, combination therapy of doxorubicin/ifosfamide has been effective [3]. Thrombocytopenia has been described in the setting of primary disease, but can be present concurrently with chronic autoimmune conditions sometimes complicating management. We report a case of metastatic uterine leiomyosarcoma that resulted in spinal cord compression and postoperative thrombocytopenia. The objective of this manuscript was to review the treatment of metastatic leiomyosarcoma to the spine and the management of postoperative thrombocytopenia.

## Case Report

An 84-year-old female presented with severe low back pain and heaviness in the legs. Referral imaging had shown a spinal lesion and associated burst fracture at the L5 level with canal stenosis. Four years earlier she was diagnosed with uterine leiomyosarcoma stage I, for which she underwent a total abdominal hysterectomy with bilateral salpingo-oophorectomy. She denied bowel or bladder dysfunction; however, she admits that upon prolonged standing she experiences increased tenderness and heaviness in her lower extremities. On physical exam she had full strength and no loss of sensation in both upper and lower extremities.

**Figure 1:**
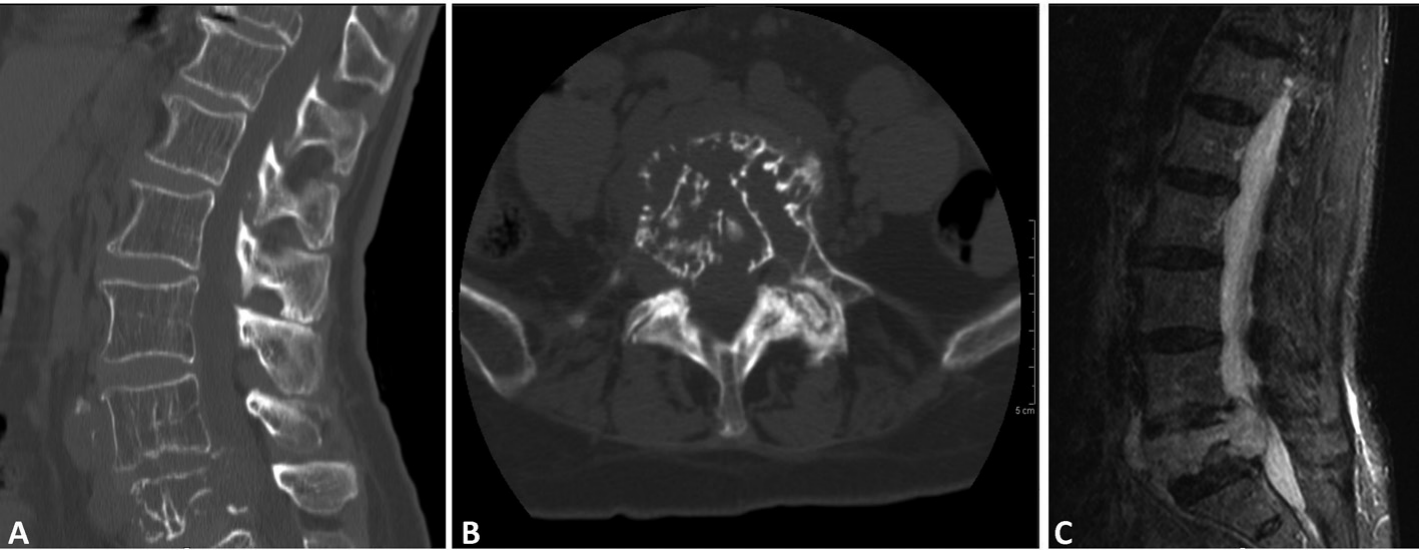
(A); (B); (C) (CT - sagittal, axial and MR T2- sagittal) soft tissue lesion extending both anteriorly and posteriorly causing canal stenosis, and destructed vertebral body, with canal involvement.

Computed tomography (CT) of the lumbar spine demonstrated a compression fracture at L5 with a soft tissue density mass causing significant canal stenosis at that level (Fig. 1A, B). An MRI scan obtained demonstrated an osteolytic lesion, compression fracture of L5, and a soft tissue mass with 1 cm retropulsion causing significant canal stenosis (Fig. 1C). AP and lateral radiographs of the T-spine showed degenerative lumbar scoliosis, and grade I L4 on L5 anterolisthesis. CT of the chest, abdomen and pelvis did not reveal evidence of neoplasm except the L5 lesion.

### Operation

**Figure 2:**
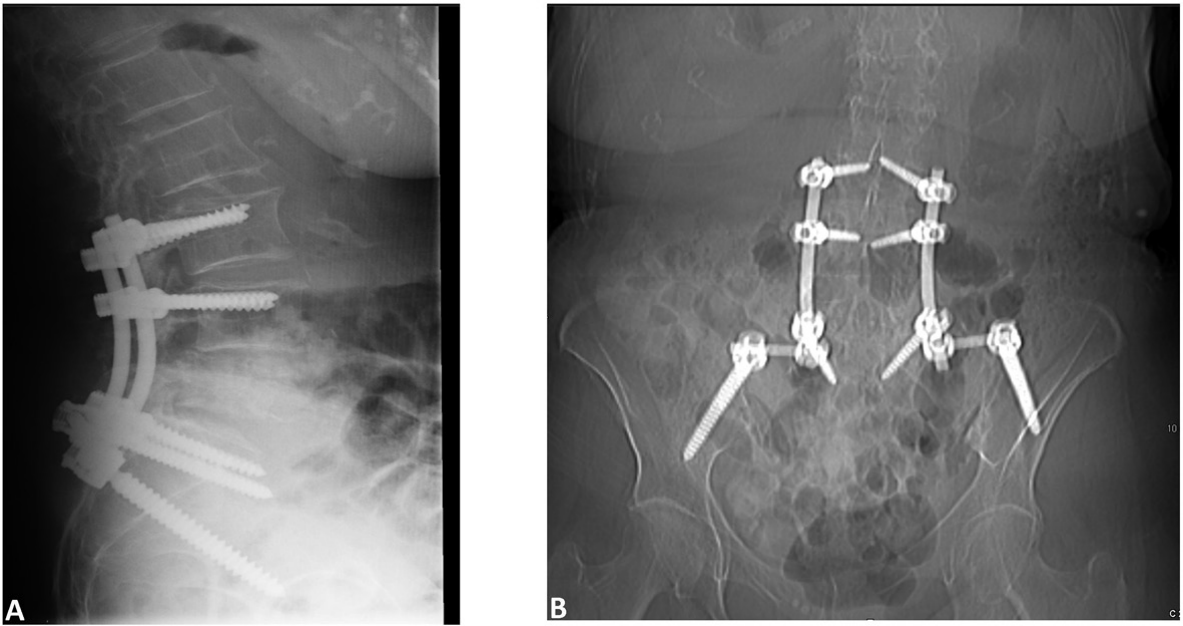
Postoperative (A) Sagittal and (B) AP images showing placement of screws.

She underwent L4-L5 laminectomy and right transpedicular approach for decompression of the burst fracture. Upon exposure of the burst fracture a soft tissue mass was palpated ventral to the thecal sac. The canal was decompressed and the sac was released of any pressure. L3 to iliac posterior spinal fusion with instrumentation was carried out with placement of bilateral pedicle screws (Fig. 2A, B). Graft arthrodesis was done using 10 mL Grafton DBM Crunch (Osteotech Inc., Eatontown, New Jersey) and 30 mL cancellous bone. Due to the oncologic nature of the procedure neither local bone nor BMP was utilized. The patient was monitored during the procedure with somatosensory evoked potentials (SSEP) without any significant changes.

### Histology

**Figure 3:**
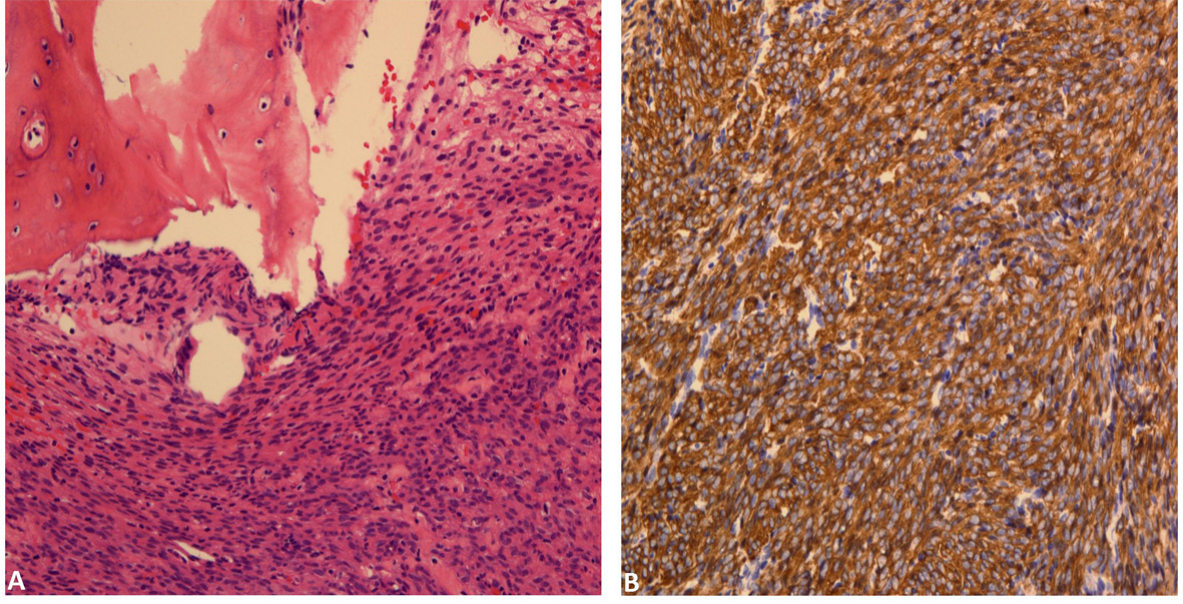
(A) Bone involved by cellular spindle cell proliferation forming fascicles with mild nuclear atypia and rare mitoses. (B). Tumor cells are diffusely positive for smooth muscle actin.

Final pathologic analysis revealed a cellular spindle cell proliferation forming fascicles with mild nuclear atypia and rare mitoses. Tumor cells invades into marrow spaces with associated bony destruction, although apparent tumor necrosis is not seen (Fig. 3A). Immunohistochemical studies showed tumor cells to be diffusely and strongly positive for smooth muscle actin (Fig. 3B), desmin and caldesmon. In addition, the morphologic features of the tumor are similar to those of the patient’s prior uterine leiomyosarcoma. Therefore, a diagnosis of metastatic uterine leiomyosarcoma involving spine is rendered.

### Post-operative course

The patient tolerated the procedure well, was ambulating on postoperative day 2 and subsequently discharged to an acute rehabilitation facility. Interestingly, prior to her starting adjuvant radiation therapy, she developed worsening thrombocytopenia despite being transfused with two apheresis packs of platelets (119,000 preoperative to 65,000 post operative/transfusion value). Despite the drop in platelets and obvious impact on hemostasis she developed no postoperative bleeding diathesis.

### Adjuvant Treatment

Radiation was recommended for her adjuvant therapy. She received 45 Gray of conformal radiation along with a 9 Gray boost. Chemotherapy was not recommended due to the low-grade nature of her pathology (low mitotic index) as well as her baseline performance status (KPS < 80%). Additionally, her underlying thrombocytopenia would have worsened further during expected bone marrow suppressive effects of cytotoxic chemotherapy.

## Discussion

The present case reveals an elderly female with metastatic uterine leiomyosarcoma to the lumbar spine. The tumor invaded the spinal canal with resultant spinal stenosis and discomfort. She was effectively decompressed and fused. Post-operatively she received adjuvant radiation therapy. Leiomyosarcomas are uncommon malignant smooth muscle tumors that occur in approximately 1% of patients with uterine cancer [1, 2]. These soft tissue tumors mostly occur in the mid to late adulthood and have a tendency to arise in the retroperitoneum, intra-abdominal space, and the subcutaneous soft tissue of the extremities [3, 4]. They usually metastasize to several sites including the abdomen, pelvis, lung, liver, and bone (including the vertebrae) [3, 5]. Like other sarcomas, when these tumors invade the spine they cause destructive lesions in the vertebral body with possible canal stenosis, which can lead to local pain and various long tract neurological symptoms. The differential diagnoses of metastatic spinal leiomyosarcomas include fibrosarcomas, malignant fibrous histiocytoma and malignant schwannoma [3, 6]. There has been an increasing number of primary leiomyosarcomas of the vertebra in relationship to EBV and HIV infection [3].

Uterine sarcomas are uncommon neoplasms with an annual incidence of approximately 0.64 to 1.7 per 100,000 women [5]. They are aggressive tumors with a poor prognosis [7-8]. Large studies have demonstrated 5-year survival rates of 20-30% overall and approximately 50% in patients with Stage I disease [2, 5]. In their review of 216 leiomyosarcoma cases including all stages Giuntoli et al reported an overall median survival of 4.02 years (range 1.3 for stage IV to 7.8years for stage I disease [2]. Gaducci et al determined a median time to recurrence for distant disease of 16 months (range 3 - 49 months) [5].

The majority of patients who recur do so within two years of initial diagnosis, and up to 90% of patients who fail show distant metastases either alone or concurrent with pelvic recurrence [5, 9]. Yet distant recurrent disease is rare as Gadducci et al found that less than 3 percent of patients (3 out of 126) presented with distant disease [5].

Bone and brain metastases are uncommon in leiomyosarcoma [3-5, 10-15]. Spinal column metastases were thought to be even less common, although in the past five years there have been less than 5 reported cases [3, 6, 16-17]. Leiomyosarcoma metastasis has been reported to occur along the entire spinal column and can be extra spinal as well as intraspinal but rarely will penetrate the dura [3, 4, 6, 11, 14-18]. Ritter et al. discussed the case of a metastatic lesion, in which the patient had paraplegia, paresthesias, originating from vertebral body, yet not invaded the epidural space [10]. These patients come to the attention of practitioners usually because of compressive symptoms, usually myelopathic symptoms of the antero-lateral cord [1, 3, 4, 6, 14-20].

Leiomyosarcomas, as evidenced in our case, can be destructive to the bone and in paravertebral lesions this most definitely contributes directly to a patient’s morbidity in terms of instability, pain and loss of function [20]. The patient in our case had lumbar involvement with distinct soft tissue metastatic leiomyosarcoma invasion and destruction of the surrounding vertebra. Additionally there was extension into the spinal canal and significant cord compression.

Leiomyosarcoma can present as primary disease to the spinal column as reported in the sacrum [14-15], Lumbar [4], thoracic [6, 18-19], and cervical spine [3, 16-17, 20]. Primary Leiomyosarcoma of the CNS, Spinal column or skull base tend to be HIV or AIDS related [3, 21], and in association with EBV [11].

Surgery is the mainstay of treatment in primary and metastatic leiomyosarcoma [2-3, 5]. The use of adjuvant chemotherapy is typically reserved for recurrent or metastatic disease [5]. Combinations of doxorubicin/ifosfamide and docetaxel/gemcitabine have given the best response rate [22]. Radiation therapy is a reasonable method in palliative therapy. Pelvic irradiation may decrease local recurrence rate without any significant impact on survival, but this is not a fail safe against distant recurrences [5].

Many women experience long-term survival in spite of metastatic disease [5], as opposed to patients with disease, apparently confined to the uterus, who may experience a clinically aggressive recurrence of tumor [2, 23].

Our patient was noted to have mild baseline thrombocytopenia, that became progressively worse in the weeks following surgery. During a literature review we found only one case of leiomyosarcoma associated thrombocytopenia from a low-grade rectosigmoid leiomyosarcoma metastatic to the heart [24]. Thrombocytopenia was reported to occur in 37% of patients with soft tissue sarcomas receiving trabectedin and doxorubicin and in 9.5% of patients with metastatic leiomyosarcoma receiving docetaxel [25-26]. Her admission platelet count was 119,000, indicative of an underlying chronic problem. The mere presence of a metastatic tumor, to the lumbar spine, with a destructive process would strongly suggest the presence of a thrombotic/sequestrative process resulting in eventual clinical thrombocytopenia, the exact etiology of which remains unclear [27]. Hematology consultation was requested as her platelet count decreased to a nadir of 47,000. Diagnostic workup including peripheral smear review, revealed thrombocytopenia with the presence of megathrombocytes. This histologic finding is consistent with the diagnosis of immune thrombocytopenia (ITP), a condition she likely had for many years. The acute decrease in platelet count perioperatively was attributed to either a benign consumptive process likely related to medications or a coincidental decrease due to fluctuating antibodies in the setting of ITP. Exhaustive evaluation did not reveal any evidence of underlying lymphoproliferative disorders or microangiopathic phenomenon.

### Conclusion

Intraspinal Leiomyosarcoma metastases are rare. Surgery is the treatment of choice. Adjuvant radiation with or without chemotherapy depending on the extent of disease can be beneficial. Thrombocytopenia can present surgical challenges, but can also be present concurrently unrelated to the primary disease.

## Abbreviations

TAHBSO: Total abdominal hysterectomy with bilateral oophorectomy
